# Navigating the rise of Fusarium meningitis: perspective on the insights and therapeutic approaches

**DOI:** 10.17179/excli2024-7737

**Published:** 2024-08-28

**Authors:** Muhammad Shaheer Bin Faheem, Omer Farooq

**Affiliations:** 1 Department of Internal Medicine, Karachi Institute of Medical Sciences, Karachi, Pakistan; 2 Shifa College of Medicine, Tameer-e- Millat University, Islamabad, Pakistan

## ⁯⁯⁯⁯⁯⁯

Meningoencephalitis can present with diagnostic challenges, and fungal agents are among the infectious agents that can cause the illness. Being one of the invasive fungal illnesses, Fusarium species have garnered a lot of attention. Major clinical symptoms of infections caused by Fusarium spp. include infections of the central nervous system (CNS), keratitis, endophthalmitis, and sino-pulmonary infections (Alavi Darazam et al., 2022[1]).

Early in 2023, two clinics in Mexico saw an epidemic of Fusarium spp. meningitis in 33 immunocompetent patients receiving epidural anesthesia. An examination of 13 patients revealed that the median time between symptom onset and hospital admission was 39 days. Despite an initial improvement on antifungal therapy, severe neurovascular consequences included aneurysm, cerebral hemorrhage, stroke, and hydrocephalus ensued. One isolate was subjected to susceptibility testing, which showed resistance to all antifungals licensed in the US but susceptibility to the experimental drug fosmanogepix. Of the four patients who survived, three were given compassionate-use fosmanogepix. Nine patients (69 percent) perished from the infection (Strong et al., 2024[3]).

Meningitis caused by Fusarium species and invasive fusariosis are becoming more common. The speed of diagnosis is increasing with the use of molecular methods. Some Fusarium species are effectively inhibited *in vitro* by novel antifungal drugs currently under development. Treatments for diseases of the central nervous system (CNS), such as Cerebrospinal fluid (CSF) filtration, may benefit from new technologies. Immunomodulatory therapies may be used in treatment since the host immune system is still crucial to healing (Strong and Ostrosky-Zeichner, 2024[4]). In the past, most patients had treatment with amphotericin B alone; more recently, some patients received treatment with voriconazole alone or in combination with amphotericin B and voriconazole. For the treatment of invasive fusariosis, current guidelines suggest using voriconazole monotherapy, lipid formulations of amphotericin B, or both in combination (Hoenigl et al., 2023[2]).

It is imperative to comprehend the clinical characteristics and management obstacles associated with Fusarium meningitis in order to deliver efficient care in the event of future outbreaks.

## Conflict of interest

None to declare.
